# Student Mental Health in UK Higher Education Institutions: Protocol for a Scoping Review of Trends, Gaps, and Research Directions

**DOI:** 10.2196/65594

**Published:** 2025-07-24

**Authors:** Nicola Cogan, Aja Murray, Emily Long, Maria Gardani, Michelle O'Toole, Menchie Leung, Stella Kyratzi, Jelena Milicev, Katie Dryburgh, Chloe Bolota, Lacey Ho

**Affiliations:** 1 Department of Psychological Sciences and Health University of Strathclyde Glasgow United Kingdom; 2 School of Philosophy, Psychology and Language Sciences University of Edinburgh Edinburgh United Kingdom; 3 School of Health and Wellbeing University of Glasgow Glasgow United Kingdom; 4 Business School University of Edinburgh Edinburgh United Kingdom; 5 Medical Research Council and Scottish Chief Scientist Office Social and Public Health Sciences Unit Medical Research Council and Scottish Chief Scientist Office Social and Public Health Sciences Unit University of Glasgow Glasgow United Kingdom

**Keywords:** mental health, United Kingdom, higher education institutions, student counselling, well-being, scoping review, evidence-based, systematic search

## Abstract

**Background:**

There is growing concern about the mental health of students in higher education institutions (HEIs) across the United Kingdom. Increasingly diverse student populations, the legacy of the COVID-19 pandemic, and variations in policies and service provision across the 4 UK nations have highlighted the need for a comprehensive understanding of student mental health. Current literature remains fragmented, with limited synthesis comparing national contexts. This protocol outlines a scoping review to systematically map the evidence base, identify key research gaps, and inform future research priorities and interventions.

**Objective:**

The primary aim is to identify and map existing research on student mental health in UK HEIs. Specific objectives include exploring how student mental health is defined and measured, identifying risk factors, assessing support systems and interventions, and highlighting gaps in research across England, Scotland, Wales, and Northern Ireland.

**Methods:**

This scoping review will follow Joanna Briggs Institute methodology and be reported in line with PRISMA-ScR (Preferred Reporting Items for Systematic reviews and Meta-Analyses extension for Scoping Reviews) guidelines. Studies will be eligible for inclusion if they (1) were conducted in the United Kingdom; (2) involved students aged 16 years and older enrolled in HEIs; (3) addressed mental health–related outcomes, definitions, or interventions; and (4) were published in English from 2005 onwards. Excluded materials include gray literature not identified during database searching. A comprehensive 3-step search strategy will be used. Initially, APA PsycInfo and MEDLINE (Ovid) will be searched, followed by a broader search across Scopus, Web of Science (Core Collection), Child and Adolescent Development Studies, and the Cochrane Library. Reference lists of the included studies will be screened for additional sources. All citations will be managed using Covidence, and study selection will involve title/abstract screening and full-text review by independent reviewers. Data extraction will include information on study design, population characteristics, mental health outcomes, measures used, interventions, and key findings. Data will be presented in tabular and narrative forms and, where appropriate, descriptive content analysis will be used.

**Results:**

As of April 2025, preliminary searches and pilot screenings have been completed. The full database search and data charting phase will commence in June 2025. The final synthesis, analysis, and stakeholder consultation are scheduled between September and November 2025. The full review will be completed by December 2025, with dissemination activities planned through early 2026.

**Conclusions:**

This review will provide a comprehensive overview of student mental health research within UK HEIs. It will highlight the key national differences, emerging themes, and underresearched areas, supporting more targeted, evidence-informed policy and practice. Limitations include language and gray literature exclusions. Nonetheless, the findings are expected to make a timely and meaningful contribution to research and intervention strategies in UK higher education.

**International Registered Report Identifier (IRRID):**

PRR1-10.2196/65594

## Introduction

### Background

Mental health challenges among students in UK higher education institutions (HEIs) have reached unprecedented levels, prompting urgent concerns across academic, clinical, and policy sectors [1-4]. Across England, Scotland, Wales, and Northern Ireland, students are reporting high and increasing rates of depression, anxiety, psychological distress, self-harm, and suicidality. In 2022, the UK Office for National Statistics reported that 37% of the students experienced moderate to severe symptoms of depression or anxiety—significantly higher than the general population of the same age group.

The COVID-19 pandemic has further intensified these difficulties, exacerbating pre-existing inequalities and placing increased pressure on both students and institutional mental health services [5,6]. Disruption to education, social isolation, financial strain, and heightened uncertainty have had a lasting impact on student well-being. However, mental health challenges are not evenly distributed across student groups. Underrepresented and marginalized populations, including mature students; students from ethnic minority backgrounds; lesbian, gay, bisexual, transgender, queer/questioning (LGBTQ+) students; international students; and neurodivergent students often face additional structural and cultural barriers that influence both their experiences of mental health and their ability to access appropriate support [7-9].

Moreover, there has been growing evidence of an increase in student suicides across UK HEIs, drawing national concern and prompting calls for systemic reform. Data from the Office for National Statistics and sector-specific analyses have highlighted that although student suicide rates remain lower than those in the general population, the absolute number of student deaths by suicide has risen in recent years. These findings have positioned suicide prevention as a growing policy and institutional priority, further underlining the urgency of building a comprehensive and evidence-informed understanding of student mental health in the United Kingdom [10-14].

At the same time, government policies aimed at widening access to HEIs, particularly among students with no family history of higher education, mature learners, and those entering university through vocational pathways, have resulted in increasingly diverse student populations. Although these policies have supported greater equity and inclusion, they have also placed greater demands on student support services, with no proportional increase in available resources [15]. Growing evidence suggests that only a relatively small proportion of students who experience mental health problems are likely to seek help [16-18], with help-seeking behaviors particularly low among international students [19-21].

Despite growing awareness of the mental health crisis in UK universities, several barriers continue to limit student access to timely and appropriate care. These include stigma, fears around confidentiality, culturally insensitive services, long waiting times, and uncertainty about where and how to seek support [22,23]. These issues are further complicated by the devolved nature of health and education policy in the United Kingdom, which has resulted in marked variations in the organization, funding, and delivery of student mental health services across England, Scotland, Wales, and Northern Ireland. For example, England has focused on NHS (National Health Service)-HEI partnerships through the University Mental Health Charter, aiming to embed a whole-university approach to mental well-being. Scotland has adopted a trauma-informed and rights-based framework within educational settings. Wales and Northern Ireland have developed distinct policy paths aligned with their devolved government priorities. These differences in the national strategy are set against diverse institutional and geographic contexts—including urban versus rural student populations, campus size, cultural diversity, and student mobility.

There are contextual disparities in the academic and student experience across the 4 nations, reflecting both systemic and demographic differences. In Scotland, the Commission on Widening Access and associated widening participation strategies have supported the inclusion of students from underrepresented groups. Wales and Northern Ireland have followed similar approaches to broaden access and attract international students [24,25]. These widening participation efforts are often implemented in smaller, more rural institutions, which may face unique capacity challenges and experience-related stressors. By contrast, English HEIs tend to have larger and more diverse student populations. International literature suggests that students in highly diverse cohorts may experience increased emotional strain and a lower sense of belonging, which can further impact mental health outcomes [26-28].

Differences in tuition models and degree structures further compound these variations. Students in England and Wales typically undertake 3-year bachelor’s programs and are required to pay full tuition fees regardless of residency status. In contrast, Scottish and Northern Irish students generally benefit from government-funded tuition and pursue 4-year degree programs. In Scotland, the first 2 years of study are broader and more interdisciplinary, with specialization occurring in later years. These structural distinctions in financial, academic, and cultural landscapes shape the student experience and may have important implications for student well-being; yet, they are rarely accounted for in mental health research or policy development.

Although international evidence on student mental health is extensive, UK-specific comparative research remains relatively underdeveloped. Some reviews have explored correlates of mental health and well-being among university students in the United Kingdom [29], and others have examined the association between financial stress and poor mental health outcomes [30]. However, these reviews do not distinguish among the distinctive sociopolitical, cultural, and institutional contexts of the 4 UK nations. There is a need for new work that actively compares and contrasts these national landscapes and explores how structural differences may affect student mental health. There is currently no comprehensive synthesis of the UK student mental health evidence base. This limits the ability of institutions, funders, researchers, and policy makers to make informed data-driven decisions, design inclusive support systems, or identify strategic priorities for future research and innovation.

A scoping review is the most appropriate methodology for addressing this gap. Unlike systematic reviews, which focus on narrowly defined research questions or interventions, scoping reviews are designed to map the breadth, nature, and range of available evidence [31]. This approach allows for a more inclusive and flexible understanding of how student mental health is conceptualized, studied, and supported across different institutional and policy contexts within the United Kingdom.

### Scoping Review Questions

#### Primary Research Question

What is the current state of research on student mental health in UK HEIs, and what are the key priority areas for future research?

#### Secondary Research Questions

##### Conceptualization and Definitions

1. How is student mental health defined, conceptualized, and framed in the UK higher education context?
2. What language, models, or theoretical frameworks are used to describe mental health and well-being?

##### Prevalence and Risk Factors

1. What is known about the prevalence and patterns of mental health conditions among UK HEI students?
2. What risks and protective factors are associated with student mental health across different demographic and social groups (eg, ethnicity, gender, sexuality, socioeconomic background, international status, disability, neurodivergence)?

##### Support Systems and Interventions

1. What forms of mental health support (eg, university services, peer interventions, NHS pathways, digital tools) are described in the literature?
2. How are these services accessed, evaluated, and experienced by students?

##### Policy and Institutional Contexts

1. How do institutional policies and service models differ across the 4 UK nations, and how are these differences reflected in the evidence base?
2. What roles do universities, governments, and other stakeholders play in shaping mental health provision?

##### Methodological Scope and Gaps

1. What study designs, populations, and measures are commonly used in research on student mental health in UK HEIs?
2. What are the key gaps in the literature in terms of geography, populations, conceptual clarity, or methodological rigor?

## Methods

The purpose of the scoping review method is to map a body of literature with the intention to illuminate the key characteristics, terms, methods, findings, interventions, and relevant gaps to inform future research [32-34]. The Joanna Briggs Institute (JBI) guidance on how to conduct a scoping review will be adhered to throughout [35].

### Ethical Considerations

As this protocol for a scoping review involves the synthesis of previously published, publicly available data and does not include human participants, ethics approval is not required. However, the review will be conducted in line with established ethical principles for secondary research, including the accurate representation of findings, acknowledgment of original sources, and transparent reporting of results following PRISMA-ScR (Preferred Reporting Items for Systematic reviews and Meta-Analyses extension for Scoping Reviews) guidelines (Multimedia Appendix 1) [36].

### Eligibility Criteria

#### Population

For the purpose of this review, the term “students” refers to individuals aged 16 years and older who are enrolled in HEIs in the United Kingdom. This includes undergraduate, postgraduate taught, and postgraduate research students across all modes of study (full-time, part-time, distance learning). There will be no upper age limit to ensure full representation of the UK HEI student population, including mature students. Students of any country of origin will be eligible, provided they are enrolled in a UK-based HEI. Studies focusing exclusively on academic staff or non-HEI student populations (eg, secondary school or further education students) will be excluded.

#### Concept

This review will include studies that specifically focus on mental health, mental illness, mental disorders, or psychological distress as they relate to students in HEIs. Eligible studies must make explicit reference to HEI students in the context of mental health–related terminology, including but not limited to depression, anxiety, suicidality, self-harm, stress, and emotional well-being. Studies that explore students’ personal understandings and lived experiences of mental health, where alternative terminology such as mental distress, madness, or culturally-specific terms are used will also be included, with attention to the language adopted by the participants themselves.

#### Context

This review will focus on research conducted within the context of UK HEIs, encompassing all 4 nations: England, Scotland, Wales, and Northern Ireland. Studies that include comparisons across these UK nations or between United Kingdom and international HEIs will also be included where relevant to the UK context.

#### Date of Publication

Eligible studies must have been published between 2005 and the present, covering a 20-year period. This time frame allows the review to capture changes in student mental health in response to evolving societal, institutional, and policy developments, including the widening participation agenda and the impact of COVID-19.

#### Types of Evidence Source

This review will include a broad range of evidence types to map the diversity of research in the field. Eligible sources include primary research studies employing qualitative, quantitative, or mixed methods designs; systematic and scoping reviews; case studies; and relevant opinion or commentary pieces that offer critical insight into student mental health in UK HEIs.

#### Language

Only studies published in English will be included, given that English is the primary language of instruction and publication in UK higher education and aligns with the context being reviewed.

### Search Strategy

A comprehensive 3-step search strategy will be employed, consistent with the JBI methodology for scoping reviews [37,38]. An initial limited search was conducted in APA PsycInfo and MEDLINE (via Ovid) to identify relevant keywords, subject headings, and search string combinations specific to student mental health within the UK higher education context. These databases were selected for their broad coverage across psychology, psychiatry, medicine, and behavioral sciences, and their relevance to multidisciplinary research in student mental health. Building on insights from this exploratory phase, the search will be expanded to include the following databases: Scopus, Web of Science (Core Collection), Cochrane Library, and Child and Adolescent Development Studies. This approach ensures broad disciplinary coverage, capturing literature from education, health, psychology, and social sciences, and supports a comprehensive mapping of the available evidence while minimizing publication bias.

The search will be limited to studies published from 2005 to the present. This 20-year time frame was selected to capture significant shifts in UK higher education and mental health policy, including the development of national strategies, service expansion within HEIs, and the impact of the COVID-19 pandemic. The time span allows for a comparative understanding of trends over 2 decades and aligns with increasing sector-wide attention to student mental health.

Search terms were developed in consultation with an academic librarian and refined based on JBI guidance [38]. A draft version of the search strategy for APA PsycInfo is provided in Multimedia Appendix 1, and this will be further adapted for use across the selected databases. Finalized search strategies for each database will be included as supplementary material with the completed review manuscript. All retrieved references will be managed using EndNote software and imported into Covidence for deduplication, screening, and selection. The review will follow the PRISMA-ScR checklist [39] to ensure transparency and methodological rigor throughout the process.

### Sources of Evidence

Prior to commencing the screening process, a calibration exercise will be conducted to ensure reliability in correctly selecting the studies for inclusion by reviewers [39]. Two levels of screening will be used to identify the sources of evidence for inclusion in the scoping review: (1) study selection—review title and abstract and (2) study screening—review the full text. The study selection will be conducted by 3 independent reviewers. Data screening, charting, and literature quality assessments will be managed using Covidence software to sift, categorize, and sort findings according to key issues and themes. Any studies identified as relevant based on the title and abstract by one or both researchers will be reviewed at full-text level. The screening process involves both reviewers evaluating the full texts of the selected studies in relation to the predetermined inclusion criteria. Where disagreement occurs between reviewers, full texts will be screened again, and discussion will take place until a consensus is met. If necessary, validation by a third reviewer will be sought. A PRISMA flowchart will be used to report the final number in the study selection process.

### Data Extraction

Data extraction will be conducted using a standardized data charting form developed in accordance with the JBI guidelines and tailored to the specific aims and research questions of this review. The form will be piloted independently by 2 reviewers on a sample of 5 studies to ensure clarity, consistency, and comprehensiveness. Any discrepancies will be discussed within the review team and amendments made to the charting form as needed. For each included study, the following data will be extracted:

1. Citation details: author(s), year of publication, journal, or source
2. Study characteristics: study design, methodology, sample size, setting, and UK nation(s) covered
3. Participant demographics: age, gender, ethnicity, student status (eg, undergraduate, postgraduate, international, first-generation, mature students)
4. Mental health focus: key concepts and definitions used; types of mental health issue or condition explored (eg, anxiety, depression, psychological distress, well-being)
5. Aims and objectives of the study
6. Measurement tools or outcome measures: instruments or self-report measures used to assess mental health (including any validated tools)
7. Key findings and themes: including prevalence data, risk and protective factors, and outcomes related to student mental health
8. Support/intervention type (if applicable): such as digital platforms, counselling services, peer support, or curriculum-integrated interventions
9. Policy and institutional context (where applicable): including links to national policies, institutional frameworks, or funding models
10. Identified gaps or research priorities: where specified by study authors
11. Funding source and any declared conflicts of interest

The extracted data will be synthesized descriptively by using a narrative synthesis approach. This will involve mapping key study characteristics and thematically analyzing findings to identify commonalities and differences in how student mental health is conceptualized, experienced, and supported across UK HEIs. Quantitative data (eg, prevalence rates, frequencies of identified risk factors) will be summarized using tables and basic descriptive statistics. Qualitative findings will be analyzed thematically by using content analysis techniques to identify recurring patterns in definitions, lived experiences, and institutional responses. Data will be mapped directly against the primary and secondary research questions. Where possible, findings will be stratified by population subgroup such as Black, Asian, ethnic minority students, neurodivergent students, LGBTQ+ students, mature learners, and international students—as well as by UK nation, to support a comparative analysis across different demographic and geopolitical contexts.

The synthesis will also highlight conceptual gaps (eg, lack of clarity or consensus around definitions of well-being, mental distress) and methodological limitations (eg, underuse of longitudinal, intersectional, participatory research designs). This will contribute to identifying evidence gaps and inform recommendations for future research directions and policy development. The process of data extraction from full-text studies will be performed by 2 independent reviewers. Discrepancies in charting will be discussed until consensus is reached; where necessary, a third reviewer will be consulted to resolve disagreements.

## Results

### Exploratory Research

An initial exploratory search was conducted in APA PsycInfo and MEDLINE (via Ovid) in March 2025 to identify relevant keywords, subject headings, and search combinations appropriate to the student mental health context within UK HEIs. This pilot search informed the refinement of the full search strategy and confirmed the feasibility of the review. Based on this initial phase, a revised and expanded search strategy was finalized in collaboration with an academic librarian.

As of April 2025, pilot testing of the inclusion and exclusion criteria has been completed by using a sample of 50 abstracts. The review team reached consensus on a clear eligibility framework aligned with the JBI methodology for scoping reviews [37,38], ensuring consistent application during the full screening process.

The full database search will be executed across APA PsycInfo, MEDLINE (Ovid), Scopus, Web of Science (Core Collection), Cochrane Library, and Child and Adolescent Development Studies in May 2025. Title and abstract screening will be completed by June 2025, followed by full-text review and data extraction in July and August 2025. The synthesis and analysis phases, including stakeholder consultation, are scheduled to take place between September and November 2025. The finalized review manuscript will be completed by December 2025, with dissemination activities planned throughout early 2026.

Data extracted from the included studies will be mapped descriptively to identify what is known about the key topics concerning student mental health in relation to the review’s research questions. The synthesis will include frequency counts for populations, concepts, and methodological characteristics, as well as tabular summaries of study features such as participant demographics, settings, mental health focus, and institutional context. Where appropriate, content analysis techniques will be applied to examine patterns in definitions, lived experiences, and support responses across UK’s 4 nations [40].

A narrative synthesis approach will be used to present key findings, supported by visual representations, including tables and charts. Data will be stratified by population subgroup (eg, mature, international, LGBTQ+, neurodivergent students) and UK nation to enable comparative analysis. This will allow for a clearer understanding of how different contexts and demographics shape mental health experiences and service access within HEIs.

A stakeholder consultation phase will follow the synthesis. This will involve presenting the findings to the broader research team and a student advisory group affiliated with the Scottish Student Mental Health Research Network (ScotSMART). Feedback will inform final refinements to the interpretation and framing of results. The results will then be disseminated to key stakeholders, including decision makers, policy developers, and academic audiences, ensuring that findings translate into actionable strategies for improving student mental health support across the United Kingdom.

### Dissemination Plan

Findings from this scoping review will be disseminated through a combination of academic and nonacademic channels to ensure wide reach, stakeholder engagement, and meaningful impact. A full-length, peer-reviewed journal paper will present the complete review outcomes, methodological approach, and key thematic findings. In parallel, a summary report will be produced for institutional stakeholders, including universities, NHS bodies, student support services, and policy organizations across England, Scotland, Wales, and Northern Ireland.

Stakeholder-targeted dissemination will include tailored reports highlighting the review’s key findings, research gaps, and actionable recommendations aligned with current priorities in the student mental health sector. These reports will be circulated to universities, health care providers, third-sector organizations, and national student mental health networks, supporting their use in practice and policy development. Academic dissemination will also include presentations at relevant national and international conferences in the fields of psychology, higher education, youth mental health, and student well-being. These may include events hosted by the British Psychological Society, Universities UK, the UK Mental Health in Higher Education Network, and global mental health research forums. Public engagement will be actively supported through a series of open-access, user-friendly outputs. These will include webinars, short-form digital summaries, and an infographic designed to communicate key insights and research gaps to a broad audience, including students, practitioners, and policy makers. Digital dissemination will also utilize institutional websites, social media platforms, and student mental health networks to enhance accessibility and visibility. To ensure real-world relevance and inclusive engagement, all dissemination materials will be co-developed with student and institutional partners, reflecting the participatory ethos that underpins this review. Input from the ScotSMART student advisory group will inform the design and framing of outputs to enhance resonance and uptake within the student community.

In line with open science principles, data management and transparency will be prioritized. All review outputs, including the finalized search strategies, data extraction forms, and synthesis tables, will be submitted as supplementary files or deposited in open-access repositories. This approach will support reproducibility, foster knowledge exchange, and maximize the long-term utility of the review findings for researchers, practitioners, and decision makers across disciplines and sectors. All dissemination materials will be co-designed with student representatives and institutional partners to ensure accessibility, relevance, and potential for real-world application. The dissemination strategy aims to inform future research, service development, and policy across UK’s higher education sector.

## Discussion

This protocol outlines a robust and comprehensive framework for conducting a scoping review aimed at mapping the existing evidence on student mental health within HEIs across the United Kingdom. The review will explore the multifaceted and contextually embedded challenges faced by students in England, Scotland, Wales, and Northern Ireland. It specifically considers issues such as financial strain, stigma, cultural, and structural barriers to help-seeking, and the compounding disadvantages faced by international, mature, neurodivergent, and underrepresented student groups. By focusing on these intersecting factors, the review seeks to provide a nuanced understanding of the mental health landscape across UK HEIs.

Building on prior reviews, which have often focused narrowly on intervention efficacy, singular mental health outcomes, or international student populations, this scoping review adopts a broader, more integrative approach. It extends the current evidence base by capturing a wider variety of study types and perspectives, incorporating literature from multiple disciplines, including education, psychology, sociology, and public health. This is particularly important in understanding the systemic, institutional, and policy-level influences on student well-being. Through its whole-system lens, this review will highlight both commonalities and divergences in how student mental health is experienced and addressed across UK’s 4 nations.

A key strength of this protocol lies in its methodological rigor, including the use of the JBI methodological guidance for scoping reviews [37,38,41] and the PRISMA-ScR checklist [36] to enhance transparency and reporting quality. The inclusion of a multidisciplinary search strategy, consultation with an academic librarian, and pilot testing of the eligibility framework further enhances the reliability and replicability of the study. Additionally, the scoping review is distinctive in its commitment to incorporating comparative analyses across United Kingdom’s devolved education systems, a relatively underexplored dimension in the existing literature.

Nevertheless, several limitations must be acknowledged. The decision to exclude gray literature may result in the omission of important contextual insights or emerging innovations that are not captured in peer-reviewed journals. Gray literature often includes institutional reports, policy briefs, or unpublished theses that could offer rich information about localized practices and lived experiences [42]. However, this exclusion is a deliberate methodological choice to ensure that the review remains manageable in scope and prioritizes high-quality, peer-reviewed evidence. The lack of standardization and variable quality in gray literature can pose challenges to data synthesis and limit the reliability of findings [43]. Another limitation is the restriction to English-language publications. Although this aligns with the dominant language of UK higher education and academic publishing, it may inadvertently exclude studies from international researchers or students working within UK HEIs who have published in other languages. However, this approach ensures feasibility within the resource constraints of the review team while maintaining focus on the linguistic context most relevant to the target population [44].

Despite these limitations, the anticipated findings of this review will make a meaningful contribution to the field. They will provide a foundation for future research by identifying conceptual and methodological gaps, including underresearched populations, neglected geographic areas, and limitations in study design such as the lack of longitudinal or intersectional analyses. Furthermore, this review will offer guidance for evaluating the effectiveness of tailored interventions and understanding how institutional structures and national policies shape student experiences. These insights will be crucial for developing evidence-informed strategies to improve mental health outcomes in HEIs across the United Kingdom.

By synthesizing a wide range of evidence and offering a cross-national comparative perspective, this review has the potential to inform policy, guide service development, and influence institutional practices. It will serve as a critical resource for stakeholders aiming to cocreate inclusive and effective mental health strategies that are responsive to the evolving needs of diverse student populations. Moreover, the integration of feedback from stakeholders, including a student advisory group within ScotSMART, will enhance the relevance, legitimacy, and applicability of the review’s conclusions.

Ultimately, this protocol establishes a timely and methodologically sound approach to understanding the state of student mental health research in the United Kingdom. In the context of growing national concern and escalating student demand for mental health services, there is an urgent need for comprehensive, contextually informed, and system-wide perspectives. This scoping review will bridge that gap by mapping the full spectrum of available evidence, highlighting where research has succeeded, and crucially, where it is still needed. In doing so, the review will support the development of coherent, equitable, and forward-looking strategies that can help transform the mental health outcomes of students across UK’s higher education sector.

## Appendix

Multimedia Appendix 1 PRISMA-ScR checklist and search strategy.

## Abbreviations

HEI: higher education institution
JBI: Joanna Briggs Institute
LGBTQ+: lesbian, gay, bisexual, transgender, queer/questioning
NHS: National Health Service
PRISMA-ScR: Preferred Reporting Items for Systematic reviews and Meta-Analyses extension for Scoping Reviews
ScotSMART: Scottish Student Mental Health Research Network
