# Supplementary material for: Student Mental Health in UK Higher Education Institutions: Protocol for a Scoping Review of Trends, Gaps, and Research Directions
Source: JMIR Res Protoc. 2025 Jul 24;14:e65594. doi: 10.2196/65594 (PMC12332455; doi:10.2196/65594)
Supplement: Multimedia Appendix 1 [file resprot_v14i1e65594_app1.docx]

# Multimedia Appendix 1

# PRISMA-ScR Checklist

| **PRISMA-ScR Checklist Item** | **Status** | **Notes** |
| --- | --- | --- |
| **Title**: Identify the report as a scoping review protocol | ✅ Complete | Title clearly identifies the review as a scoping review protocol. |
| **Abstract**: Structured summary | ✅ Complete | Abstract includes background, objectives, eligibility, information sources, and synthesis plan. |
| **Rationale**: Describe rationale for the review | ✅ Complete | Comprehensive justification for a scoping review approach provided. |
| **Objectives**: State the questions and objectives | ✅ Complete | Primary and secondary questions are clearly defined. |
| **Protocol and Registration**: State whether registered | ✅ Complete | The protocol is registered: https://doi.org/10.21203/rs.3.rs-3292219/v1 |
| **Eligibility Criteria**: Define inclusion/exclusion criteria | ✅ Complete | Detailed criteria for population, concept, context, and study types are provided. |
| **Information Sources**: Describe databases and coverage dates | ✅ Complete | Multiple databases listed with date range (2005–present). |
| **Search Strategy**: Present draft search strategy | ✅ Complete | Search strategy for APA PsycInfo is provided in Multimedia Appendix 2. |
| **Selection of Sources**: Describe screening process | ✅ Complete | Title/abstract and full-text screening by independent reviewers using Covidence. |
| **Data Charting Process**: Describe data extraction approach | ✅ Complete | Piloted standardised data charting form with dual reviewers. |
| **Data Items**: List variables and definitions | ✅ Complete | Full list of extracted variables provided. |
| **Critical Appraisal of Sources**: If conducted, describe methods | ✅ Not applicable | No critical appraisal planned, in line with JBI guidance for scoping reviews. |
| **Synthesis of Results**: Describe planned synthesis method | ✅ Complete | Narrative synthesis and descriptive mapping described, with stratification where appropriate. |
| **Ethics and Dissemination**: Address ethics approval and dissemination plans | ✅ Complete | Ethics exemption explained; dissemination strategy includes academic, stakeholder, and public. |
| **Funding**: Describe sources of funding and funder role | ✅ Complete | Funding from Royal Society of Edinburgh (RSE #2509) declared. |

# Sample Search Strategy for APA PsycInfo (Ovid)

APA PsycInfo (via Ovid)

1. (student* adj3 ("mental health" or "mental illness" or "mental distress" or "psychological distress" or anxiety or depression or "self harm" or suicidality)).ti,ab.
2. (higher education or university or universities or college or HEI).ti,ab.
3. (UK or "United Kingdom" or England or Scotland or Wales or "Northern Ireland").ti,ab.
4. 1 and 2 and 3
5. Limit to English language
6. Limit to publication year 2005–2025
